# Thioredoxin Reductase 1 Is a Highly Immunogenic Cell Surface Antigen in *Paracoccidioides* spp., *Candida albicans*, and *Cryptococcus neoformans*

**DOI:** 10.3389/fmicb.2019.02930

**Published:** 2020-01-09

**Authors:** Fabiana Freire Mendes de Oliveira, Verenice Paredes, Herdson Renney de Sousa, Ágata Nogueira D’Áurea Moura, Juan Riasco-Palacios, Arturo Casadevall, Maria Sueli Soares Felipe, André Moraes Nicola

**Affiliations:** ^1^Faculty of Medicine, University of Brasília, Brasília, Brazil; ^2^Department of Molecular Microbiology and Immunology, Johns Hopkins Bloomberg School of Public Health, Baltimore, MD, United States; ^3^Karan Technologies Research and Development, Brasília, Brazil; ^4^Department of Microbiology, Institute of Biomedical Sciences II, University of São Paulo, São Paulo, Brazil; ^5^Department of Dermatology, Faculty of Medicine, University of São Paulo, São Paulo, Brazil; ^6^Graduate Program in Genomic Sciences and Biotechnology, Catholic University of Brasília, Brasília, Brazil

**Keywords:** *Cryptococcus neoformans*, *Candida albicans*, *Paracoccidioides lutzii*, thioredoxin reductase, cell wall, antibodies

## Abstract

The increasing number of immunocompromised people has made invasive fungal infections more common. The antifungal armamentarium, in contrast, is limited to a few classes of drugs, with frequent toxicity and low efficacy pointing to the need for new agents. Antibodies are great candidates for novel antifungals, as their specificity can result in lower toxicity. Additionally, the immunomodulatory activity of antibodies could treat the underlying cause of many invasive mycoses, immune disfunction. In a previous comparative genomics study, we identified several potential targets for novel antifungals. Here we validate one of these targets, thioredoxin reductase (TRR1), to produce antibodies that could be useful therapeutic tools. Recombinant TRR1 proteins were produced by heterologous expression in *Escherichia coli* of genes encoding the proteins from *Candida albicans*, *Cryptococcus neoformans*, and *Paracoccidioides lutzii*. These proteins were then used to immunize mice, followed by detection of serum antibodies against them by ELISA and western blot. A first set of experiments in which individual mice were immunized repeatedly with TRR1 from a single species showed that all three were highly immunogenic, inducing mostly IgG1 antibodies, and that antibodies produced against one species cross-reacted with the others. In a second experiment, individual mice were immunized three times, each with the protein from a different species. The high titers of antibodies confirmed the presence of antigenic epitopes that were conserved in fungi but absent in humans. Immunofluorescence with sera from these immunized mice detected the protein in the cytoplasm and on the cell surface of fungi from all three species. These results validate TRR1 as a good target for potentially broad-spectrum antifungal antibodies.

## Introduction

Fungal diseases are estimated to affect around a billion people each year, leading to 1.5 million deaths (Bongomin et al., 2017). The incidence for these diseases is bound to remain high, as they frequently affect people that are rendered immunocompromised by advances in Medicine such as organ transplantation, immunossupression and chemotherapy. In addition to opportunistic infections, systemic mycoses caused by primary pathogens such as *Paracoccidioides lutzii* are also important causes of morbidity and mortality in regions such as Latin America (Fortes et al., 2011; Martinez, 2015) Broad-spectrum treatment options for these diseases is restricted to drugs from a few chemical families acting primarily against membrane and cell wall targets, such as azoles, polyenes, and echinocandins (Nett and Andes, 2016). Other antifungal classes such as the pyrimidine analog flucytosine and ergosterol biosynthesis-inhibiting allylamines have much narrower spectra (Sable et al., 2008; Fuentefria et al., 2018). Price and availability in the developing world are a major concern for several of these drugs, as is the increase in resistance (Sable et al., 2008; Chang et al., 2017).

There is thus a dire need for new and effective antifungal drugs, an area of research and technological development in which some advances have been made (Del Poeta and Casadevall, 2012). In a previous work from our group (Abadio et al., 2011), we identified potential targets for antifungals using comparative genomics. We identified ten genes as high-priority targets using several criteria, such as that the target genes should be (a) present in most or all of the most important pathogenic fungi, (b) absent from (or significantly different in) the human genome, (c) essential or important for the survival of the fungi of interest, and (d) located in a part of the fungal cell that is accessible to antifungal agents. Among these genes is *TRR1*, which encodes a thioredoxin reductase. This enzyme is crucial for cellular redox homeostasis and is essential in *Candida albicans* (Abadio et al., 2011) and *Cryptococcus neoformans* (Missall and Lodge, 2005).

Considering that immune dysfunctions are frequent in cases of invasive mycoses, antibodies might be advantageous because they would add to the inhibition of the target a second therapeutic mechanism: immunomodulation (Kullberg et al., 2014; Rodrigues et al., 2016). The objective of this work, then, was to validate TRR1 as a target for antibody development. We found that this protein is highly immunogenic, has conserved epitopes and can be found in the cell wall, which suggest it might be a successful immunotherapy target.

## Materials and Methods

### Microbial Strains and Culture

*Escherichia coli* BL21 (DE3) and DH5α strains were grown in LB medium at 37°C and conserved with 50% of LB and 50% of glycerol at −80°C. Fungal strains H99 (*C. neoformans*) and SC5314 (*C. albicans*) were grown in solid YPD for 48 h and conserved in 50% of liquid YPD and glycerol 50% at −80°C. *P. lutzii* strain Pb01 was maintained by passage every 7 days in Fava-Netto medium; cells were collected for experiments at 5 days after passaging.

### Mammalian Cell Culture and Protein Extraction

Human embryonic kidney (HEK293) cells (Gibco) were thawed and cultured in Freestyle F17 expression medium (Gibco) at 37°C, 5% CO_2_. For total protein extraction, cells were pelleted at 200 × *g*, washed with PBS and resuspended in cold RIPA buffer (20 mM Tris–HCl, 140 mM NaCl, 1% Triton X-100, 0.5% SDS, 1 mM EDTA, and 1 mM phenylmethylsulfonyl fluoride, pH 7.5), then vortexed for 30 s, incubated on ice for 30 min and centrifuged at 14,000 × *g*, 10 min. The supernatant containing soluble proteins was stored at −20°C for further analysis.

### Mice

Six-week-old female BALB/c mice were used to perform the immunizations. These experiments were made in the Animal Facility of the Johns Hopkins Bloomberg School of Public Health, Johns Hopkins University, or in the Bioassays Laboratory of the Catholic University of Brasília. The experiments were done according to the approved protocols MO15H134 (Johns Hopkins University) and 018/13 (Catholic University of Brasília).

### Recombinant Protein Production, Purification, and Quantification

*TRR1* genes were codon-optimized and chemically synthesized by two different companies, Epoch Biolabs and Genscript. In both cases, the genes were cloned into the *Xho*I and *Nde*I sites of the pET-21a vector (Novagen), which was purified with a Qiagen plasmid Midiprep kit following manufacturer’s instructions. The vector was transformed in *E. coli* BL21 DE3 to produce the recombinant proteins, which were induced with 0.25 mM IPTG when cultures were at optical densities between 0.4 and 0.6. They were purified by affinity chromatography on HisPur^TM^ Cobalt Chromatography Cartridges (Thermo Fisher), with imidazole elution. Protein preparations were analyzed by polyacrylamide gel electrophoresis (Bio-Rad), concentrated by ultrafiltration (Millipore Centriprep^TM^) and quantified by spectrophotometry. For some experiments we also used as negative control an unrelated, his-tagged recombinant protein that was prepared as part of a different project (Moura et al., manuscript in preparation). This protein (*P. lutzii* HSP90) was produced, purified, concentrated, and quantified with a similar strategy.

### Murine Immunization

Five groups of one to three animals each were separated according to the condition of the immunization: (1) Control, injected only with PBS in adjuvant. (2) Animals immunized only with *C. albicans* TRR1. (3) Animals immunized only with *P. lutzii* TRR1. (4) Animals immunized only with *C. neoformans* TRR1. (5) Animals immunized sequentially with TRR1 from the three different species (*C. albicans* – *P. lutzii* – *C. neoformans*). The animals were immunized with a subcutaneous injection in the back of the neck with an emulsion of 50% Freund’s adjuvant in PBS containing 50 μg of protein. Three immunizations were made in each mouse, with a 2-week interval between immunizations. The first immunization was made with complete and the others with incomplete Freund’s adjuvant. Sera were obtained before all immunizations (and 2 months after the last one) by retro-orbital bleeding using heparin capillary tubes in mice under isoflurane anesthesia.

### Western Blot

Purified recombinant proteins (144 ng per lane), HEK293 protein extracts (80 μg per lane) and a molecular weight marker (PageRuler Prestained Protein Ladder – Thermo Fisher) were separated by electrophoresis on denaturing 10% polyacrylamide gels. The proteins were then transferred to a nitrocellulose membrane (GE Healthcare Life Sciences), which was blocked with 5% skim milk in TBS (20 mM Tris–HCl, 150 mM NaCl, pH 7.4). The membranes were then incubated with a 1:6.000 dilution of the sera from immunized mice and, after washing, with a secondary antibody to mouse light chains conjugated with HRP (Jackson Immuno Research). The membranes were then incubated with SuperSignal^TM^ West Pico PLUS Chemiluminescent Substrate (Thermo Fisher) and imaged on a ChemiDoc system (Bio-Rad).

### ELISA

TRR1 proteins diluted to 10 μg/mL were used to coat polystyrene plates. After blocking with 1% BSA in PBS, dilutions of the sera from the immunized mice were incubated for 1 h at 37°C. In some of the experiments, we used as secondary antibody a combination of alkaline phosphatase-conjugated goat anti-mouse IgG, IgA, and IgM (Southern Biotech) in a 1:1000 dilution also for 1 h at 37°C. In other experiments, we used isotype-specific (IgA, IgG1, IgG2a, IgG2b, IgG3, and IgM) AP-conjugated secondary antibodies (Southern Biotech). Bound antibodies were detected using p-nitrophenyl phosphate (Sigma) as a substrate, with absorbance measured in a plate spectrophotometer at 405 nm. In some experiments we also included as negative control wells in which the TRR1 proteins were substituted for an unrelated his-tagged protein, to detect antibodies against the tag used for TRR1 purification.

### Immunofluorescence

Fungal cells were fixed with 4% paraformaldehyde and washed with PBS. The serum of mice that had been immunized sequentially with TRR1 from all three fungal species was used as primary antibody in a dilution of 1:100, followed by Alexa Fluor^®^ 488 conjugated anti-mouse IgG antibody (ThermoFisher Scientific) diluted 1:100. After washing and mounting, the cells were imaged in a Zeiss AxioObserver Z1 microscope equipped with a 63× objective. Z-stacks were collected and deconvolved using a constrained iterative algorithm with the Zeiss ZEN software.

## Results

### Production of TRR1 Proteins From *C. albicans, C. neoformans*, and *P. lutzi*i

**FIGURE 1:**
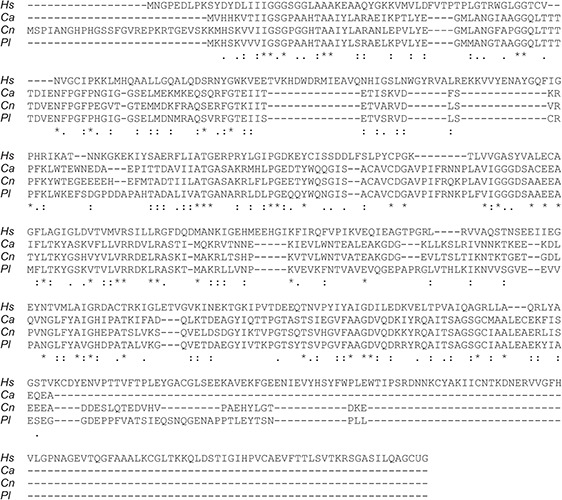
Alignment of TRR1 sequences. TRR1 sequences were obtained from online databases and aligned using Clustal Omega (Madeira et al., 2019). *Hs, Homo sapiens; Ca, C. albicans*; *Cn, C. neoformans*; *Pl, P. lutzii*. The symbols indicate full conservation (^∗^), strong similarity (:), and weak similarity (.) in each position.

**FIGURE 2:**
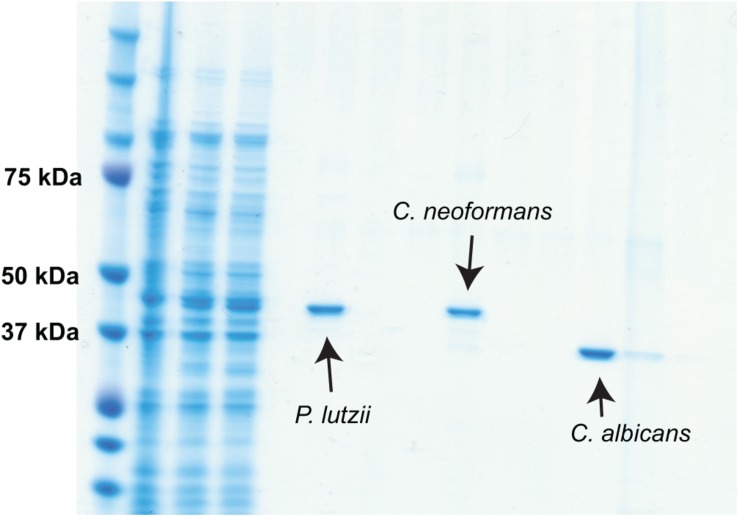
Production of his-tagged TRR1 proteins. Genes encoding *TRR1* from *C. albicans*, *C. neoformans*, and *P. lutzii* were chemically synthesized and cloned into bacterial expression vectors. After transformation in *E. coli* and induction, the recombinant TRR1 proteins were purified by affinity chromatography and analyzed by SDS-PAGE.

Sequences encoding TRR1 from *P. lutzii*, *C. albicans*, and *C. neoformans* were obtained from FungiDB (Basenko et al., 2018), whereas their human homolog was obtained from UniProt. A Clustal Omega alignment of them (Figure 1) shows three regions with reasonable variation among the different fungal species interspersed with highly conserved regions. TRR1 proteins from the three species are between 63 and 76% identical among each other, but have only 21 to 24% identity with the human thioredoxin reductase. Genes encoding each of the TRR1 proteins were chemically synthesized and cloned in pET21 vectors for heterologous expression in *E. coli*. As shown in Figure 2, we were able to produce highly purified TRR1 proteins from all three species. This experiment was repeated twice with vectors produced from two different companies, with no discernible difference in the proteins produced.

### Immunization With TRR1 Proteins Generate High Titers of Cross-Reactive Antibodies

**FIGURE 3:**
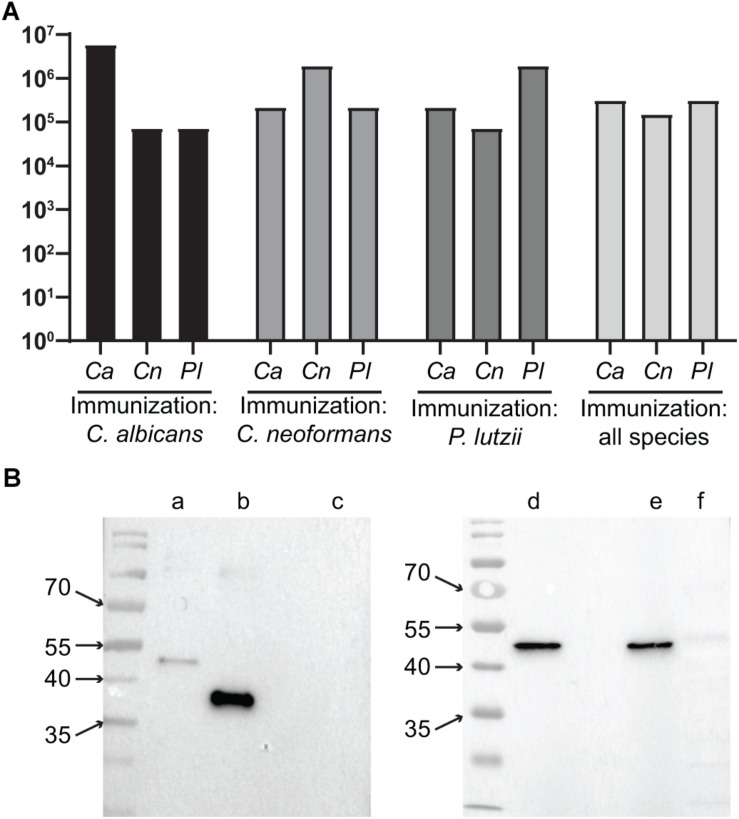
Heterospecific and homospecific antibodies to TRR1. **(A)** Titers of homospecific and heterospecific antibodies in animals immunized with TRR1. Each animal was immunized three times with TRR1 from a single species or with the protein from all three species, one at a time. Two months after the last injection their sera were collected and used in ELISA experiments in which the plates were coated with TRR1 from each of the three fungal species. Bars represent the titers of antibodies (IgA + IgG + IgM) against each one of those species. **(B)** Sera from mice that were immunized with all three fungal species in sequence were used in western blot experiments to detect binding to recombinant *C. albicans* (lane **b**), *C. neoformans* (lanes **a**,**d**), and *P. lutzii* (lane **e**) TRR1 proteins, as well as the protein extract of the human cell line HEK293 (lanes **c**,**f**).

We used the purified recombinant TRR1 proteins to immunize mice in different strategies. Initially, one group was immunized with only *C. albicans* recombinant TRR1 protein, another with only TRR1 from *C. neoformans* and a third with *P. lutzii* TRR1. Each of these mice were immunized three times, and the titers of antibodies (IgA + IgG + IgM) recognizing the recombinant proteins from all three species measured by ELISA. As shown in Figure 3A, TRR1 proteins from all three species induced titers of more than 1:5.904.900 of antibodies that bound to the species used as an immunogen (homospecific antibodies). *C. albicans* was the most immunogenic protein, followed by *P. lutzii* and *C. neoformans*. The sera also contained antibodies that were cross-reactive with TRR1 proteins from other species than those that were used as immunogen (heterospecific antibodies). Heterospecific titers varying from 1:72.900 to 1:656.100, being higher in animals immunized with *C. albicans* TRR1 and lower in those immunized with the *C. neoformans* protein. We next immunized animals with a different strategy. They were immunized three times as the other ones were, but with TRR1 from a different species each time. As shown in Figure 3A, anti-TRR1 titers in these mice varied from 1:72.900 to 1: 5.904.900. The same sera from mice immunized with all three TRR1 proteins was used in western blot experiments with the recombinant proteins and a protein extract from a human cell line, HEK293 (Figure 3B). TRR1 proteins from all three species were recognized by the antibodies, but not the human homolog.

To determine which immunoglobulin isotypes were induced by immunization with TRR1 proteins, we repeated these experiments with isotype specific secondary antibodies. As shown in Supplementary Figure S1, the isotype with highest titers in all animals was IgG1, with variable titers for other IgG isotypes and IgM and little IgA. As all TRR1 proteins had a 6x-His tag, we also included as negative control an unrelated his-tagged protein to measure the amount of antibodies that recognized the purification tags instead of the TRR1 protein. The geometric mean of the titers of IgG1 antibodies to this protein in mice immunized with TRR1 proteins was 3.5 × 10^4^, approximately 200 times lower than the geometric mean titer of IgG1 to the immunogens (7.0 × 10^6^).

Given the very high titers and cross-reactivity of antibodies to TRR1, we analyzed the protein sequences using T cell and B cell epitope prediction tools. The NetMHCIIpan tool (Jensen et al., 2018) predicted four different regions that contained peptides that probably bind strongly to mouse MHC class II (Supplementary Figure S2). Two of these regions are highly conserved and predicted to occur in all three species. BepiPred-2.0, a linear B cell epitope prediction tool, suggested the existence of 10–12 epitopes in each of the three sequences. Nine of these are predicted to occur in all three species, of which one is identical in all of them, six have 50–90% identity among the species and two have less than 50% identity. Of the nine regions with linear epitopes in all three species, six were also predicted as part of conformational epitopes in the *C. neoformans* TRR1 crystal structure using ElliPro (Ponomarenko et al., 2008). Supplementary Figure S3 shows the location of some of the epitopes on the TRR1 sequence.

### Antibodies to TRR1 Bind to the Fungal Cell Surface

**FIGURE 4:**
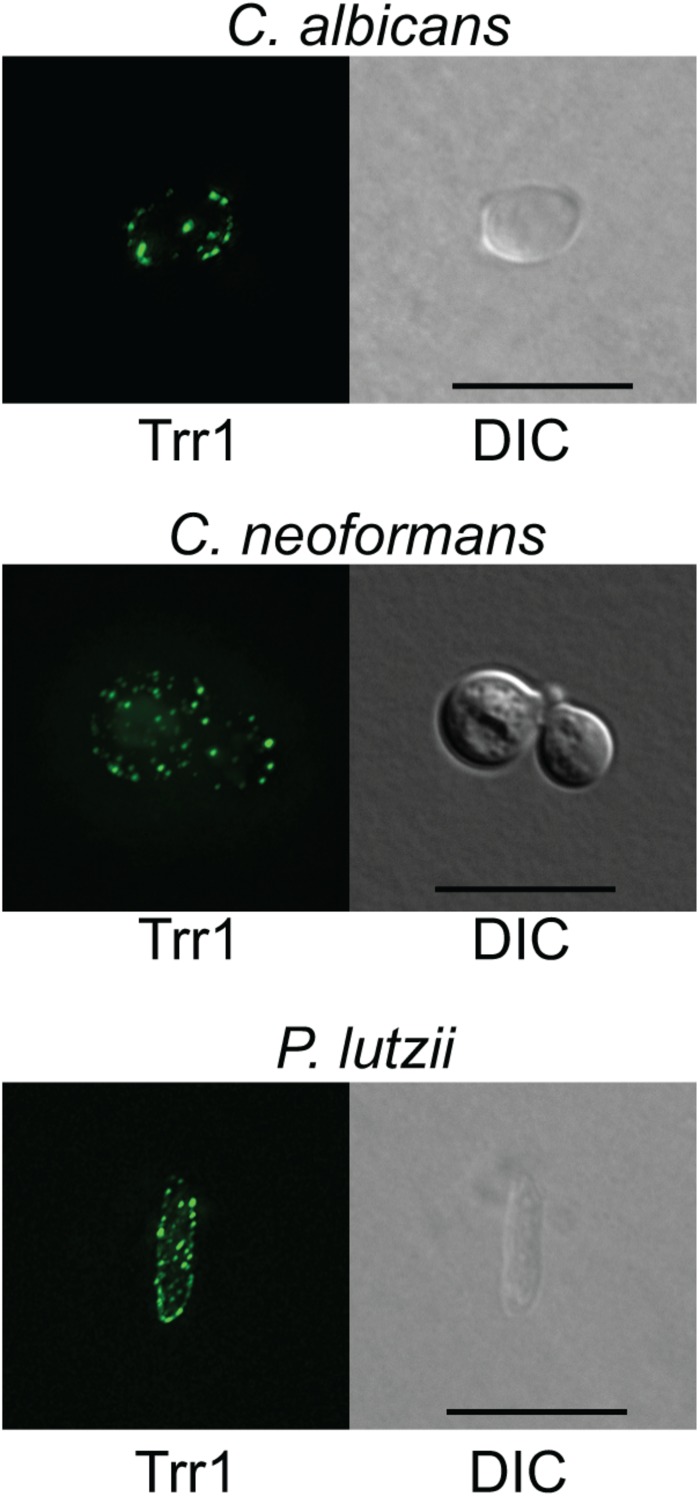
Immunofluorescence with anti-TRR1 sera. *C. albicans, C. neoformans*, and *P. lutzii* yeast cells were fixed, permeabilized and incubated with sera from mice that had been immunized with the TRR1 proteins from all three species. Bound antibodies were detected with a fluorescently labeled secondary antibody. Positive spots can be observed on the surfaces of all three cell types.

We used the sera from mice that were sequentially immunized with TRR1 proteins from all three species in immunofluorescence experiments with *C. albicans*, *C. neoformans*, and *P. lutzii* yeast cells. As shown in Figure 4, we observed significant binding of antibodies in the antiserum to the cell surface in all three types of yeast cells, in a punctate pattern.

## Discussion

The increase in the life expectancy of immunocompromised patients afforded by modern medicine has come with a higher incidence of opportunistic fungal infections like invasive aspergillosis and candidiasis. In addition to that, the number of cases of severe mycoses associated with AIDS and infections by primary fungal pathogens such as *Paracoccidioides* spp. and *Histoplasma capsulatum* still remain high (Travassos and Taborda, 2017). Considering the dire need for new antifungal drugs, almost a decade ago we seized the opportunities brought by genomics studies to discover new drug targets (Abadio et al., 2011). Our focus in one of these targets, TRR1, has already resulted in the discovery of new small molecule antifungal candidates (Abadio et al., 2015). TRR1 might also be a target for auranofin, an antirheumatic drug that displayed interesting properties in studies that aimed to repurpose it as an antifungal (Siles et al., 2013; Fuchs et al., 2016; Thangamani et al., 2017; Wiederhold et al., 2017). In this work we made the first steps in validating this target for antibody-based therapies as well.

Antibodies play important roles in the immune response to fungi (Casadevall and Pirofski, 2012), and also have great potential for immunotherapy of fungal diseases (Zhou and Murphy, 2006; Taborda and Nosanchuk, 2017). The fact that antibodies are naturally found in mammals and the exquisite specificity of the humoral immune response can result in drugs with lower toxicity and lead to less adverse events in comparison with existing therapies. In addition to inhibiting their targets, the immunomodulatory effects of antibodies can also contribute to curing mycoses that frequently happen in immunodepressed individuals (Ravikumar et al., 2015). Two antibody therapies for fungal diseases made it to clinical development: efungumab, an antibody fragment targeting *C. albicans* HSP90 with positive results in phase III clinical trials (Pachl et al., 2006) that was discontinued and 18B7, a murine antibody to the *C. neoformans* capsule that gave promising results in a phase I clinical trial (Larsen et al., 2005).

In addition to these two antibodies that made it to clinical development, several other antibodies have been proposed as candidates for immunotherapy of fungal diseases (Nicola et al., 2018). An anti-β-glucan monoclonal antibody (mAb 2G8) was protective in animal models of invasive mycoses (Rachini et al., 2007), consistent with the protection afforded by a β-glucan vaccine (Torosantucci et al., 2005). Interestingly, other antibodies had as targets proteins that are not traditionally associated with the cell wall like TRR1, such as the cytosolic molecular chaperones HSP90 (Matthews et al., 1991) and HSP60 (Guimarães et al., 2009) and a histone-like protein (Nosanchuk et al., 2003). In *C. neoformans* (Missall and Lodge, 2005) and *Fusarium graminearum* (Fan et al., 2019), GFP-tagged TRR1 was localized in the cytoplasm and mitochondria, the sites in which its enzymatic function is carried out. Our immunofluorescence experiments, however, showed the protein is distributed in a punctate pattern on the surface of the three fungi studied. This pattern has been associated before with increased antifungal activity for an antibody to the *C. albicans* cell wall (Casanova et al., 1990), which is promising for antifungal therapy. Consistent with this extracellular presence of TRR1, it was found in *C. neoformans* extracellular vesicles (Rodrigues et al., 2008) and on the extracellular matrix of mature *C. albicans* biofilms (Martínez et al., 2016). This suggests that cell wall TRR1 would indeed be a good target for therapeutic antibodies, which cannot penetrate intact fungal cells. It also poses an interesting question of whether it is “moonlighting” (Jeffery, 2018) in the cell wall by playing a specific physiologic role there.

Our animal tests showed that TRR1 was highly immunogenic, corroborating a previous study made with *C. albicans* TRR1 (Godoy et al., 2016). Moreover, the immunization led to the production of mostly IgG1 and other IgG isotypes, the expected response to protein antigens (Vidarsson et al., 2014). These are also the isotypes most commonly used to generate therapeutic antibodies, given that the vast majority of therapeutic antibodies are IgG. More importantly, the protein from different species seemed to share B (and probably T) cell epitopes that led to high titers of broadly cross-reactive antibodies, considering the significant phylogenetic distance between the basidiomycete *C. neoformans* and the ascomycetes *C. albicans* and *P. lutzii*. In line with this high immunogenicity and cross-reactivity, a study in which sera from mice infected with *Coccidioides posadasii*, *C. albicans*, and *P. brasiliensis* were incubated with an array containing recombinant *Saccharomyces cerevisiae* proteins detected antibodies that were induced by fungal infection and cross-reacted with baker’s yeast TRR1 (Coelho et al., 2015). Strikingly, TRR1 was one of only 16 out of 4,800 different *S. cerevisiae* proteins present on the array that were recognized by antibodies in sera from animals infected with all three fungi, which together with our data indicate that TRR1 is an immunodominant antigen. This has interesting applications for TRR1 as a therapeutic antibody target, suggesting a higher possibility of a broad-spectrum drug. Given that mice immunized with *C. albicans* TRR1 were more resistant in an animal model of invasive candidiasis (Godoy et al., 2016), these findings also raise the question of the role immune responses against cell wall TRR1 play in antifungal immunity.

## Data Availability Statement

All datasets generated for this study are included in the article/Supplementary  Material.

## Ethics Statement

Experiments with animals were reviewed and approved by the Catholic University of Brasília Commision for Ethics on the Use of Animals and the Johns Hopkins University Animal Care and Use Committee.

## Author Contributions

FO, AN, MF, and AC conceptualized and designed the experiments. FO, HS, ÁM, JR-P, and VP acquired, analyzed, and interpreted data from experiments, which were supervised by AC, AN, and MF. FO and AN drafted the manuscript, which was revised by all authors.

## Conflict of Interest

The authors declare that the research was conducted in the absence of any commercial or financial relationships that could be construed as a potential conflict of interest.
