# Supplementary figures and images for: Thioredoxin Reductase 1 Is a Highly Immunogenic Cell Surface Antigen in Paracoccidioides spp., Candida albicans, and Cryptococcus neoformans
Source: Front Microbiol. 2020 Jan 9;10:2930. doi: 10.3389/fmicb.2019.02930 (PMC6964600; doi:10.3389/fmicb.2019.02930)

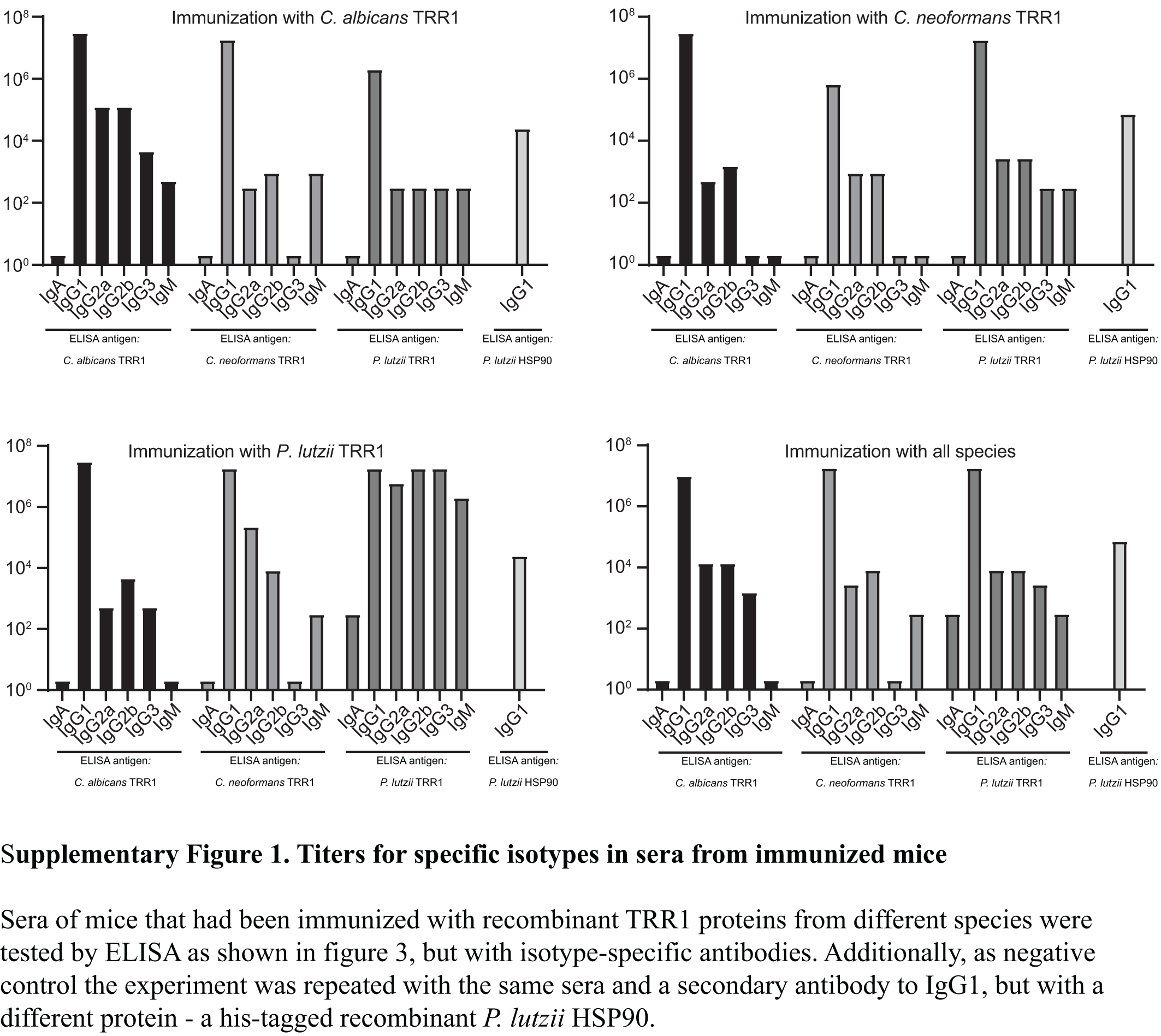

Supplement: Supplementary file 1 [file Image_1.TIF]

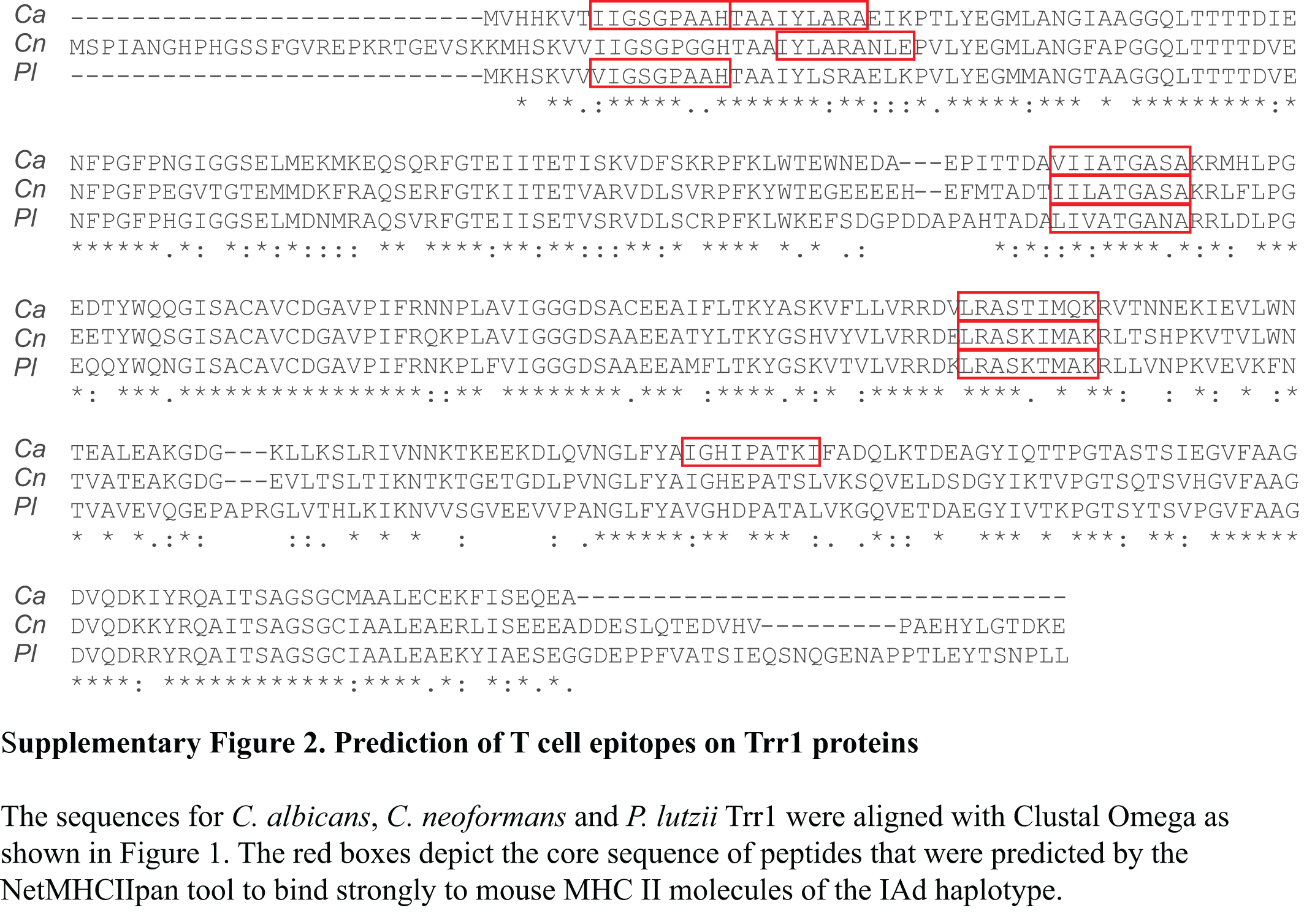

Supplement: Supplementary file 2 [file Image_2.TIF]

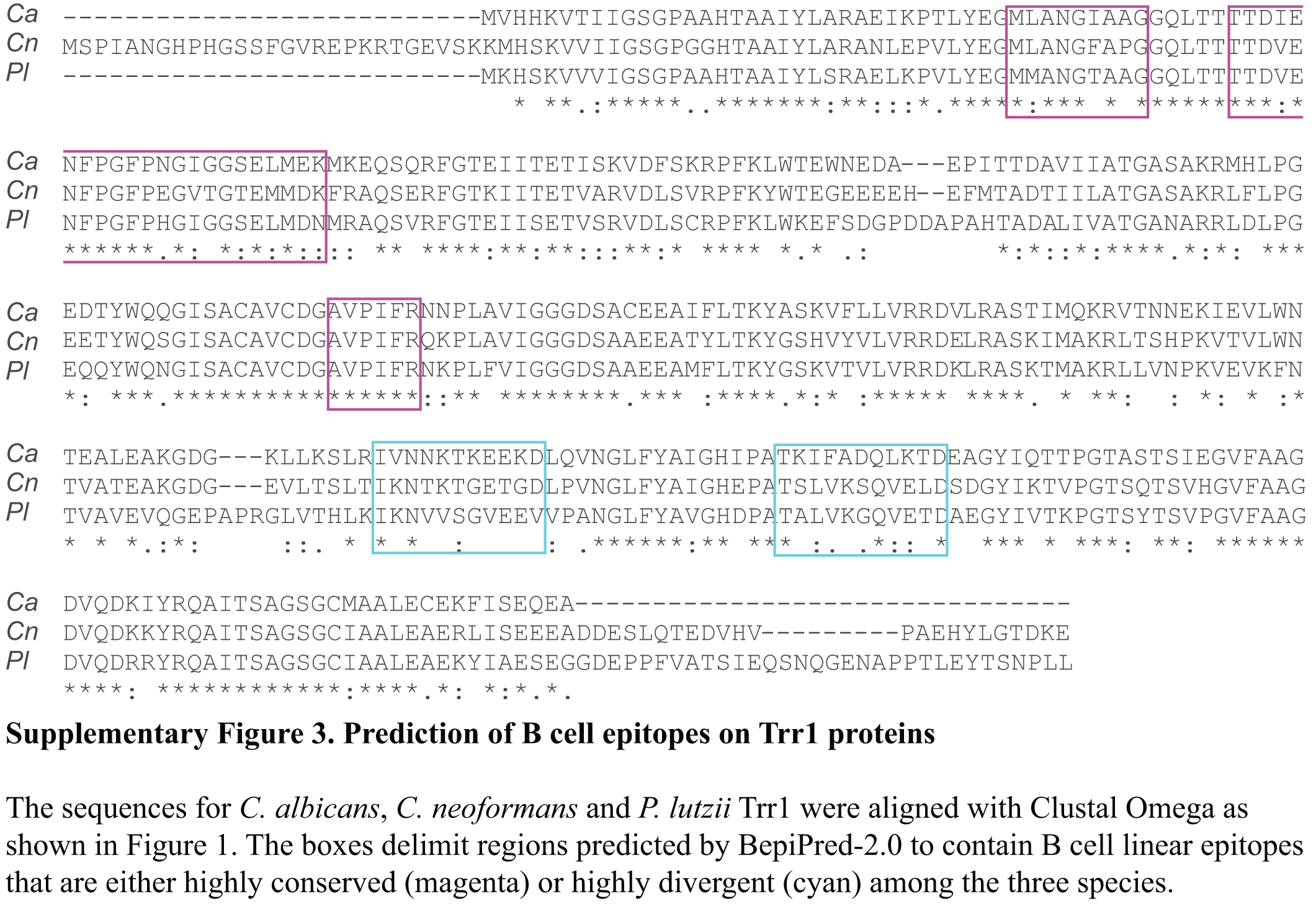

Supplement: Supplementary file 3 [file Image_3.TIF]
